# Incidence of biopsy-proven giant cell arteritis (GCA) in South Australia 2014–2020

**DOI:** 10.3389/fmed.2023.1173256

**Published:** 2023-04-20

**Authors:** Jem Ninan, Carlee Ruediger, Kathryn A. Dyer, Thomas Dodd, Rachel J. Black, Suellen Lyne, Ernst M. Shanahan, Susanna M. Proudman, Susan Lester, Julian McNeil, Catherine L. Hill

**Affiliations:** ^1^Discipline of Medicine, University of Adelaide, Adelaide, SA, Australia; ^2^Rheumatology Unit, Northern Adelaide Local Health Network, Modbury, SA, Australia; ^3^Rheumatology Unit, The Queen Elizabeth Hospital, Woodville, SA, Australia; ^4^SA Pathology, Adelaide, SA, Australia; ^5^Rheumatology Unit, Royal Adelaide Hospital, Adelaide, SA, Australia; ^6^School of Medicine, Flinders University, Bedford Park, SA, Australia; ^7^Rheumatology Unit, Southern Adelaide Local Health Network, Bedford Park, SA, Australia

**Keywords:** incidence, giant cell arteritis (GCA), South Australia, biopsy, positive

## Abstract

**Objective:**

To determine the incidence of biopsy proven giant cell arteritis (GCA) in South Australia.

**Methods:**

Patients with biopsy-proven GCA were identified from pathology reports of temporal artery biopsies at state-based pathology laboratories, from 1 January 2014 to 31 December 2020. Incidence rates for biopsy-proven GCA were calculated using Australian Bureau of Statistics data for South Australian population sizes by age, sex, and calendar year. Seasonality was analyzed by cosinor analysis.

**Results:**

There were 181 cases of biopsy-proven GCA. The median age at diagnosis of GCA was 76 years (IQR 70, 81), 64% were female. The estimated population incidence for people over 50 was 5.4 (95% CI 4.7, 6.1) per 100,000-person years. The female: male incidence ratio was 1.6 (95% CI 1.2, 2.2). There was no ordinal trend in GCA incidence rates by calendar year (*p* = 0.29). The incidence was, on average, highest in winter, but not significantly (*p* = 0.35). A cosinor analysis indicated no seasonal effect (*p* = 0.52).

**Conclusion:**

The incidence of biopsy-proven GCA remains low in Australia. A higher incidence was noted compared to an earlier study. However, differences in ascertainment and methods of GCA diagnosis may have accounted for the change.

## Introduction

Although giant cell arteritis (GCA) is the commonest primary vasculitis in the elderly, it is still an uncommon condition. Confirmation of the clinical diagnosis of GCA is challenging as almost all the available diagnostic tests have significant limitations. Temporal artery biopsy is the reference test, despite its sensitivity which varies from 39 (1)–90% (2). Imaging of the temporal artery by ultrasound is not as widely accepted for diagnosis in Australia as it is in Europe (3).

A recent meta-analysis of GCA incidence showed an overall incidence of 10/100,000 over 50 years and confirmed the highest incidence in Scandinavian countries, with decreasing incidence in Americas, the remainder of Europe and Oceania (4). It also confirmed the significant association with latitude (4). All epidemiological studies have confirmed the higher incidence in women than men by ratio of 2:1 (4).

Although the exact mechanism of disease is unknown, infectious triggers have been postulated (5). This has led to studies of seasonal variation in GCA incidence. A seasonal increase in GCA incidence has been reported in some studies with peaks in the summer months in the USA (6) and Europe (7); however, a previous study did not confirm this in Australia (8). This is supported by a recent comprehensive meta-analysis (9) which did not confirm a seasonal onset for GCA. In addition, some studies have demonstrated cyclical trend showing an incidence peak every 7 years (10, 11).

We previously determined the incidence of biopsy-proven GCA in South Australia until 2011, to be significantly lower than that from other geographic regions. Therefore, the aim of the current study was to determine the current incidence of GCA in South Australia.

## Materials and methods

### Ascertainment of biopsy-proven GCA cases

All pathology reports of patients who underwent temporal artery biopsy were identified from the electronic data base of SA Pathology laboratories from 1 January 2014, to 31 December 2020. SA Pathology is the largest public pathology service in South Australia accounting for approximately 75% of temporal artery biopsy processing in South Australia.

All pathology reports of temporal artery biopsies during this time period were reviewed and patients with biopsy-proven GCA were identified. Patients were defined as having biopsy-proven GCA if this diagnosis was made by the reviewing pathologist on the diagnostic report. The pathology reports were also reviewed by the authors.

In addition, we were able to identify the number of patients who underwent temporal artery biopsy at private laboratories (ClinPath Laboratory and Australian Clinical Labs) in South Australia between 2017 and 2020.

### Statistical analysis

Epidemiological data were analyzed using Stata v16.1 (StataCorp LLC, Texas, USA) v16.1. Incidence rates per 100,000 person years were estimated by Poisson regression using the South Australian population data for each relevant year, stratified by age and gender, as an offset (12). The estimated population incidence was then adjusted by simulation to account for the underestimation related to lack of complete population coverage by SA Pathology.

A non-parametric trend test (nptrend) was used to test for ordinal trends in incidence rates by calendar year.

A cosinor analysis was performed to calculate seasonal trends in incidence using “R” software (version R—3.6.2) (13) and the “season” library (14, 15). This analysis fits a cosine curve to the monthly data available for the years January 2014 to December 2020.

Data from a previous South Australian study were used to determine trends in incidence rates, age (grouped into 5-year categories) and sex over the periods 1 January 1992, to 31 July 2011 and 1 January 2014 to 31 December 2020 (current study, SA Pathology data only). These comparisons were performed using standardized (adjusted) rates calculated using the combined South Australian population age, and sex distribution of both studies as the standard population.

### Ethics approval

This study has ethics approval at Central Adelaide Local Health Network Human Research Ethics Committee and Southern Adelaide Local Health Network Human Research Ethics Committee.

## Results

Temporal artery biopsies were performed on 858 patients between 1 January 2014 and 31 December 2020 and processed by SA Pathology. One-hundred and eighty-one patients with biopsy-proven GCA were identified giving a biopsy positive rate of 21.8%. The median age at diagnosis of GCA was 76 years (IQR 70, 81), 64% were female. The estimated population incidence for people over 50 was 4.0 (95% CI 3.5, 4.6) per 100,000-person years based on the SA Pathology data. During the period 2017–2020, 163 biopsies were performed at private laboratories. This means that during the same period 502/665 (75%) were performed at SA Pathology during this period. The incidence estimate, adjusted for incomplete biopsy ascertainment, was 5.4 (95% CI 4.7, 6.1) per 100,000-person years.

**FIGURE 1:**
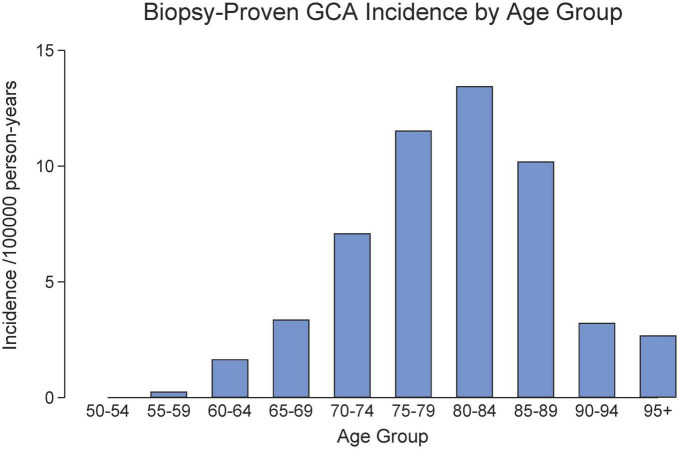
Age distribution of biopsy-proven GCA in South Australia (2014–2020).

**FIGURE 2:**
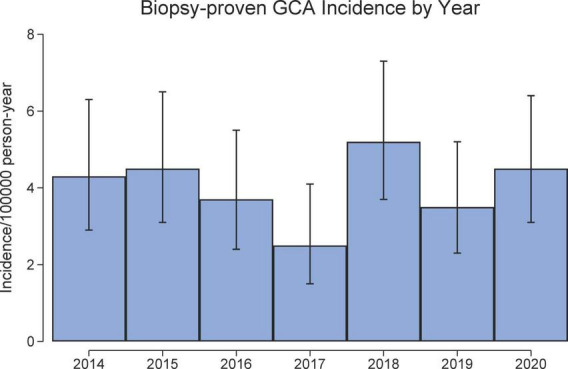
Incidence of biopsy-proven GCA in South Australia (2014–2020).

The incidence increased with each age decade with peak incidence in 70–89-year age group (Figure 1). The female: male incidence ratio was 1.6 (95% CI 1.2, 2.2). There was no ordinal trend in GCA incidence rates by calendar year (*p* = 0.29; Figure 2). The incidence rates were, on average, highest in winter, but not significantly so (*p* = 0.35). A cosinor analysis also indicated no seasonal effect (*p* = 0.52).

When compared with data from a prior study of GCA incidence in South Australia, the standard incidence rate of biopsy-positive GCA in South Australia increased by approximately 25% for the period 2014–2020 (current study) compared to the prior study period between 1991–2011 (reference), incidence rate ratio (IRR) 1.25 (95% CI 1.04, 1.50), *p* = 0.017. This was primarily attributable to an increase in the incidence rate in males (IRR 1.46, 95% CI 1.07, 1.99) between the two study periods, which was not evident in females (IRR 1.17, 95% CI 0.93, 1.47). This difference could not be ascribed to an increased referral of males for biopsy, as the proportion of males referred was comparable between the two study periods (*p* = 0.92).

The peak age-group for a positive biopsy was 75–79 years in both study groups, and there was no overall difference in the age category distributions (ordinal probit regression, *p* = 0.37).

## Discussion

The incidence of biopsy-proven GCA in Australia is low compared to many other Western countries.

In a recent meta-analysis of the incidence of GCA, the pooled incidence (not limited to biopsy positive cases) was 10.00 (9.22, 10.78) cases per 100,000 people over 50 years old (4). The current South Australian estimate of 5.4 cases per 100,000 population is substantially lower which is at least in part contributed to by the fact that this analysis only includes biopsy-proven cases.

Our incidence was lower in comparison to a similar study of biopsy proven GCA from North West Spain by Gonzalez-Gay et al. (16) which reported an annual incidence rate of 10.13 per 100,000 population aged 50 years and older, with mean age ± SD at the time of diagnosis being 75.0 ± 6.9 years. The female to male ratio was 1.6:1 in comparison to their study in which there was no statistical difference in incidence between men and women. The reasons for the lower incidence rate in our population is not entirely clear and cannot be attributed to geographical reasons alone. A recent study from New Zealand reported their incidence rate for biopsy-proven GCA at 10.5 per 100,000 people over the age of 50 (17).

The current estimate of biopsy-proven GCA incidence was higher than that observed in a prior South Australian study (8). Several factors may have contributed to this, including ascertainment bias as we changed from manual searching to computerized searching between estimates; and change in practice such that more suspected cases undergo temporal artery biopsy, rather than treatment based on clinical suspicion alone. However, incidence rates have been reported to have decreased by 0.80 per 100,000 people per year over time between 1987 and 2017 in Scandinavian countries presumed to be due to increased immigration from low incidence countries (4).

Unlike some previous studies, we found no evidence of seasonal trends similar to the study from North West Spain (16). This was also confirmed by a recent comprehensive meta-analysis (9).

Of interest, there was no change in GCA incidence in 2020. There was no reduction in referral for temporal artery biopsies in 2020 as may have been expected in the pandemic. In South Australia, incidence of COVID-19 infection was extremely low in 2020; however, social distancing was closely adhered to. The latter led to the reduction in viral infections in general in South Australia which did not appear to have any effect on GCA incidence rates in 2020. This may raise some doubt about the viral trigger hypothesis of GCA. On the contrary, several studies have demonstrated an increased incidence of GCA during 2020 (18–20).

The mean age at GCA diagnosis has remained stable over a 30-year period despite tighter recommendations for blood pressure and lipid control over this time period. This is in contrast to a study by Kermani et al. (21) who observed an increase in average age at GCA diagnosis from 75 years in the 1950s to 79 years in the 2000s.

The strength of this study is that we were able to systematically capture most temporal artery biopsies (75%) and hence most cases of biopsy-proven GCA within a confined population. One of the limitations of the study is that we adjusted for the missing temporal artery biopsies based on the assumption that the biopsy positivity rate from other pathology services is similar to that at SA Pathology. Our study is confined to biopsy-proven GCA and does not include patients with GCA diagnosed by imaging such as PET scan, CT scanning or imaging, nor patients who were treated clinically as GCA either without having a temporal artery biopsy or with a negative biopsy result.

## Conclusion

In conclusion, the incidence of biopsy-proven GCA in this South Australian population based study has increased but remains low compared to other Western populations. The increased incidence observed since the previous analysis may be related to changes in case ascertainment and patterns of temporal artery biopsy utilization.

## Data availability statement

The original contributions presented in this study are included in the article/supplementary material, further inquiries can be directed to the corresponding author.

## Ethics statement

The studies involving human participants were reviewed and approved by Central Adelaide Local Health Network Human Research Ethics Committee (HREC) Number 2009145. Written informed consent for participation was not required for this study in accordance with the national legislation and the institutional requirements.

## Author contributions

JN: data collection and writing the manuscript. SLe: data analysis and figures. CH: editing, data review, and data collection. JM, SP, RB, and ES: editing and advice. KD: database and data collection. CR: data collection. TD: pathology reports data base search. All authors contributed to the article and approved the submitted version.
